# Physical activity on executive function in sedentary individuals: Systematic review and meta-analysis of randomized controlled trials

**DOI:** 10.1371/journal.pone.0294251

**Published:** 2023-12-07

**Authors:** Shudong Tian, Zhide Liang, Fanghui Qiu, Xianliang Wang

**Affiliations:** 1 School of Physical Education, Shandong University, Jinan, 250061, China; 2 Department of Physical Education, Qingdao University, Qingdao, 266071, China; University of Turin: Universita degli Studi di Torino, ITALY

## Abstract

Physical activity has been demonstrated to promote cognitive performance. However, the relationship between physical activity and executive function (EF) in sedentary individuals is not fully understood. This meta-analysis examined the impact of physical activity on EF in sedentary individuals and evaluated potential moderators of the relationship between physical activity and EF. In accordance with the PRISMA guidelines, the electronic databases MEDLINE, Embase, PsycINFO and Web of Science were searched. Included studies had to report sedentary individuals randomized to either a physical activity group or a control group. Subgroup analyses of EF sub-domains, exercise prescription and age were conducted alongside the overall meta-analysis. Thirteen RCT studies were included, with a total of 752 participants. Results showed a small to moderate beneficial effect of physical activity on EF (SMD = 0.24, 95% CI 0.08 to 0.40). In subgroup analysis by EF sub-domains, physical activity enhanced inhibitory control (SMD = 0.38, 95% CI 0.12 to 0.63) and working memory (SMD = 0.22, 95% CI -0.05 to 0.49), but not cognitive flexibility (SMD = 0.11, 95% CI -0.18 to 0.41). Interventions with an intervention length > 12 weeks improved overall EF (SMD = 0.26, 95% CI 0.06 to 0.46), but intervention length ≤ 12 weeks did not (SMD = 0.20, 95% CI -0.08 to 0.47). Interventions with session time ≥ 45 minutes improved overall EF (SMD = 0.47, 95% CI 0.22 to 0.77), but session time < 45 minutes did not (0.17, 95% CI -0.11 to 0.44). Physical activity improves EF for older adults (age ≥ 60 years) (SMD = 0.25, 95% CI 0.08 to 0.42), but not for younger individuals (age < 60 years) (SMD = 0.17, 95% CI -0.25 to 0.59). Overall, physical activity has a beneficial effect on EF in sedentary individuals, although the influence may be domain specific and influenced by exercise prescription and age. These findings have practical implications for those seeking to improve EF in sedentary individuals through physical activity.

## Introduction

Executive function (EF) is a top-down, higher-order cognitive functions that are responsible for reasoning, planning, regulating, controlling information processes and behaviors. Core EFs consist of inhibitory control, working memory and cognitive flexibility. The capacity to suppress strong or inappropriate impulses from within or from the outside is referred to as inhibitory control. Working memory is the ability to store information in the mind for short periods of time and is essential for reasoning and problem solving. Cognitive flexibility represents the ability to adjust flexibly to changing needs or priorities [1]. EF is relevant to multiple aspects of life, including physical and mental health as well as social, cognitive and psychological development, and can predict quality of daily life and health [2]. A systematic review included 12 cross-sectional studies that investigated the relationship between sedentary behavior and EF [3]. The studies concluded that sedentary behavior was associated with poorer EF across the lifespan. In addition, physical activity is not only good for physical health but also produces positive effects on EF [4,5]. However, to our knowledge, there is currently no systematic review that examines the effects of physical activity on brain health using improvements EF sub-domains in sedentary individuals as a research endpoint.

Sedentary behavior (SB) and physical activity are two separate but related lifestyles that take up all the waking hours of a day. Sedentary behavior was defined as any form of waking activity that involves sitting or lying down and results in energy expenditure of less than 1.5 metabolic equivalents (METs) [6]. Accumulating evidence showed that high levels of sedentary behavior are linked to an increased risk of chronic conditions, such as obesity [7], type 2 diabetes, cardiovascular disease [8,9], and mental illness [10]. Despite this, nearly 67% of adults worldwide are still sedentary for more than 8.5 hours during waking hours [11]. Physical activity is characterized as any movement of the body that elevates energy expenditure beyond that at rest [12]. Being more active and less sedentary is essential for maintaining a healthy lifestyle because of its beneficial effects on skeletal [13], cardiovascular [7] and metabolic levels [14]. A previous meta-analysis study have found small effect of physical activity on inhibitory control in old sedentary individuals [15]. Notably, this study only included specific exercise prescriptions, cognitive components (i.e., inhibitory control) and age groups (i.e., older adults) [15]. However, research has found that physical activity characteristics (duration, frequency and intensity) and different cognitive components contribute to differences in the impact of physical activity on people’s cognitive performance. In addition, characteristics of participants, such as age, physical fitness and cognitive ability, were also found to affect the relationship [16].

Individual demographic characteristics may be a moderator of the effect of physical activity on EF in sedentary individuals [17]. For example, age may be an important moderating factor due to differences in the maturation of the prefrontal cortex at different ages [18]. Studies have found that EF is usually associated with the healthy functioning of the prefrontal cortex [19]. In addition, the results of the meta-analysis showed that older adults could achieve greater EF benefits after physical activity compared to other adult groups [20]. This finding suggests greater adaptive reserve in older people experiencing significant cognitive recession. However, the generalizability of these findings to sedentary individuals is yet to be established. In addition to individual characteristics, the relationship between physical activity and EF in sedentary individuals may also be influenced by exercise prescriptions, including session time, intervention length and exercise frequency [21,22]. Due to the dose-response relationship, the volume of physical activity may be another important moderator [23]. Therefore, the benefits of EF may vary depending on the session time. Experimental studies have shown that 20 minutes of physical activity have a greater impact on EF than 45 minutes and 10 minutes [24]. However, some studies report still inconsistent results in the performance of EFs in sedentary individuals after 40 minutes of physical activity [25,26]. Last but not least, EF sub-domains may play an important moderating role, each with a different degree of sensitivity to physical activity [20]. Observational studies have shown that physical activity in individuals is positively correlated with EF, with particularly pronounced effects on inhibitory control and working memory [27]. However, some studies report inconclusive evidence regarding the cognitive benefits of physical activity in healthy populations. For instance, Verburgh et al. (2014) [18] found no significant impact of physical activity on cognitive function in cognitively healthy older adults. Additionally, Singh et al. (2019) [28] revealed insufficient evidence to support the notion that physical activity improves cognitive performance in children.

In summary, there is currently limited evidence on the effects of physical activity interventions on the cognitive performance of sedentary individuals. Therefore, the purpose of this meta-analysis review was to examine the effects of physical activity on EF in sedentary individuals. Furthermore, we evaluated its potential moderation by examining relevant moderators. Specifically, determine the moderating effects of EF sub-domains (i.e., inhibitory control, working memory and cognitive flexibility), exercise prescription (i.e., intervention length, exercise frequency and session time) and individual characteristics (i.e., age) on physical activity to improve EF in sedentary individuals.

## Materials and methods

The methodology employed in this meta-analysis was carried out in strict accordance with the guidelines established by the Preferred Reporting Items for Systematic Reviews and Meta-Analyses (PRISMA) [29] as well as the Cochrane Collaboration Handbook Statemen [30]. This review was prospectively registered with PROSPERO (registration number CRD42023433776).

### Literature search strategy

Sample two review authors (S.D. and Z.D.) conducted electronic article searches in the Web of Science, PsycINFO, Embase and MEDLINE databases between January 2000 and January 2023. The titles and abstracts of the relevant studies were carefully reviewed by two separate authors, with any discrepancies resolved through mediation by a third author (X.L.) until consensus was reached. All possible search terms (from MeSH) contained in each search string are concatenated using the Boolean operators “AND” and “OR”. The combination of four sets of search terms were used to locate the studies, respectively the physical activity terms (“physical activity (PA)” OR “aerobic exercise” OR “chronic exercise” OR “exercise” OR “training”) AND sedentary behavior terms (“sedentary behavior” OR “sedentary lifestyle” OR “screen time” OR “sitting time”) AND EF terms (“executive function” OR “executive control” OR “cognition” OR “working memory” OR “inhibitory control” OR “cognitive flexibility” OR “cognitive function”) AND selected terms regarding study designs (“randomized controlled trial”). To ensure the thoroughness of this study, a comprehensive search of the Web of Science was conducted to include any potentially eligible studies from previous meta-analyses. All database specific search queries are available in **S1 Appendix**.

### Inclusion and exclusion criteria

Inclusion criteria were established for the selection of studies in this review: (1) Studies to be included in the review must be randomized controlled trials (RCT). (2) The study must focus on the effects of physical activity interventions on EF in sedentary individuals. (3) Participants must be sedentary or inactive healthy individuals. (4) Articles examining the relationship between physical activity and/or sedentary behavior and outcomes in at least one sub-domain of EF (inhibitory control, working memory, and cognitive flexibility) must be reported. (5) Studies must report the results of pre- and post-intervention testing of EF. (6) All included studies are peer-reviewed published articles written in English. Regarding the exclusion criteria, we did not include conference abstracts in the study because their quality reporting was not feasible [31]. Secondly, studies with multiple behavioral health interventions (e.g., diet programs or sleep programs combined with physical activity) were excluded because no independent effects of physical activity on EF could be obtained for sedentary behavioral health individuals. The eligibility criteria for the relevant studies are shown in Table 1.

**Table 1:** Eligibility criteria for relevant studies.

Criteria	Inclusion	Exclusion
Populations	Sedentary individuals in the study and without other medical conditions	Have serious mental health or cardiovascular disease
Interventions	physical activity	Diet and sleep plan combined with exercise
Comparators	Stretching control	No control group
Outcomes	Executive function	Studies not include outcomes or with incomplete outcomes
Time	At least 7 times	Less than 7 times
Study designs	RCTs	Other study designs, secondary analysis

### Data extraction

Two authors performed independent extraction of study background information (first name of author, year of publication, study site), sample characteristics, physical activity protocol, and EF sub-domains of eligible studies. To calculate the pre-post intervention differences, we followed the guidelines outlined in the Cochrane Collaboration Handbook, using Mean and Standard Deviation (SD) values (SD _difference_ = SQRT (SD _pre_^2^ + SD _post_^2^)– 2 * CRC * SD _pre_* SD _post_, Mean _difference_ = Mean _post_−Mean _pre_), with a correlation coefficient (CRC) of 0.5 [30].

### Risk of bias assessment

Two review authors independently assessed the methodological quality and risk of bias of included studies using the Cochrane Collaboration Risk of Bias tool [30] and the Strengthening the Reporting of Observational Studies in Epidemiology (STROBE) guidelines [32]. The Cochrane Collaboration guidelines identified seven domains, including random sequence generation, allocation concealment, blinding of participants, blinding of outcome assessments, incomplete outcome data, selective outcome reporting, and other sources of bias. The potential risk of bias in each domain was assessed as “low risk”, “high risk”, or “unclear bias”. In instances where there were discrepancies between the two authors conducting the quality evaluation, a third author (F.H.) was consulted to facilitate resolution and ensure consensus. The quality assessment data are summarized in **S1 Fig in S1 Appendix.**

### Statistical analysis

Considering the variations in the outcomes and indicators of EF measures utilized across the included studies, standardized mean differences (SMD) were calculated by means of the pre- and post-intervention means, and weights were given by their inverse variance. The magnitude of the effect sizes was assessed using Cohen’s d values, with SMD values of < 0.2, 0.2 ≤ SMD < 0.5, and 0.5 ≤ SMD < 0.8 indicating small, moderate, and large effect sizes, respectively [30]. Quantitative pooled analysis based on a fixed-effects model, and a random-effects model if heterogeneity exists. To assess the degree of statistical heterogeneity in the meta-analysis, we employed the *I*^2^ statistic and *p*-value. Specifically, *I*^2^ values of 25%, 50%, and 75% were used to indicate mild, moderate, and high levels of heterogeneity, respectively [33]. Sensitivity analyses was performed to identify the sources of heterogeneity, and we utilized the literature-by-deletion method to perform a sensitivity analysis. Potential bias in the study is assessed using an adjusted funnel plot, which can be visualized and used to detect the presence of any dominant type of potential bias, such as publication bias, selective reporting, or other bias. We also assessed publication bias with the Egger bias test [34].

Following a meta-analysis of overall EF, a series of subgroup analyses were conducted in order to explain any observed heterogeneity or to explore potential moderating effects. Our variables of interest included EF sub-domains (inhibitory control, working memory, and cognitive flexibility), exercise frequency (≤ 2 times/week or ≥ 3 times/week), intervention length (≤ 12 weeks or > 12 weeks), session time (≤ 45 min or > 45 min), and age (young-old/ ≤ 60 years or > 60 years). All the above analysis sequences were performed in STATA version 15.1 (Stata, Corp, College Station, Texas, USA).

## Results

**Fig 1:**
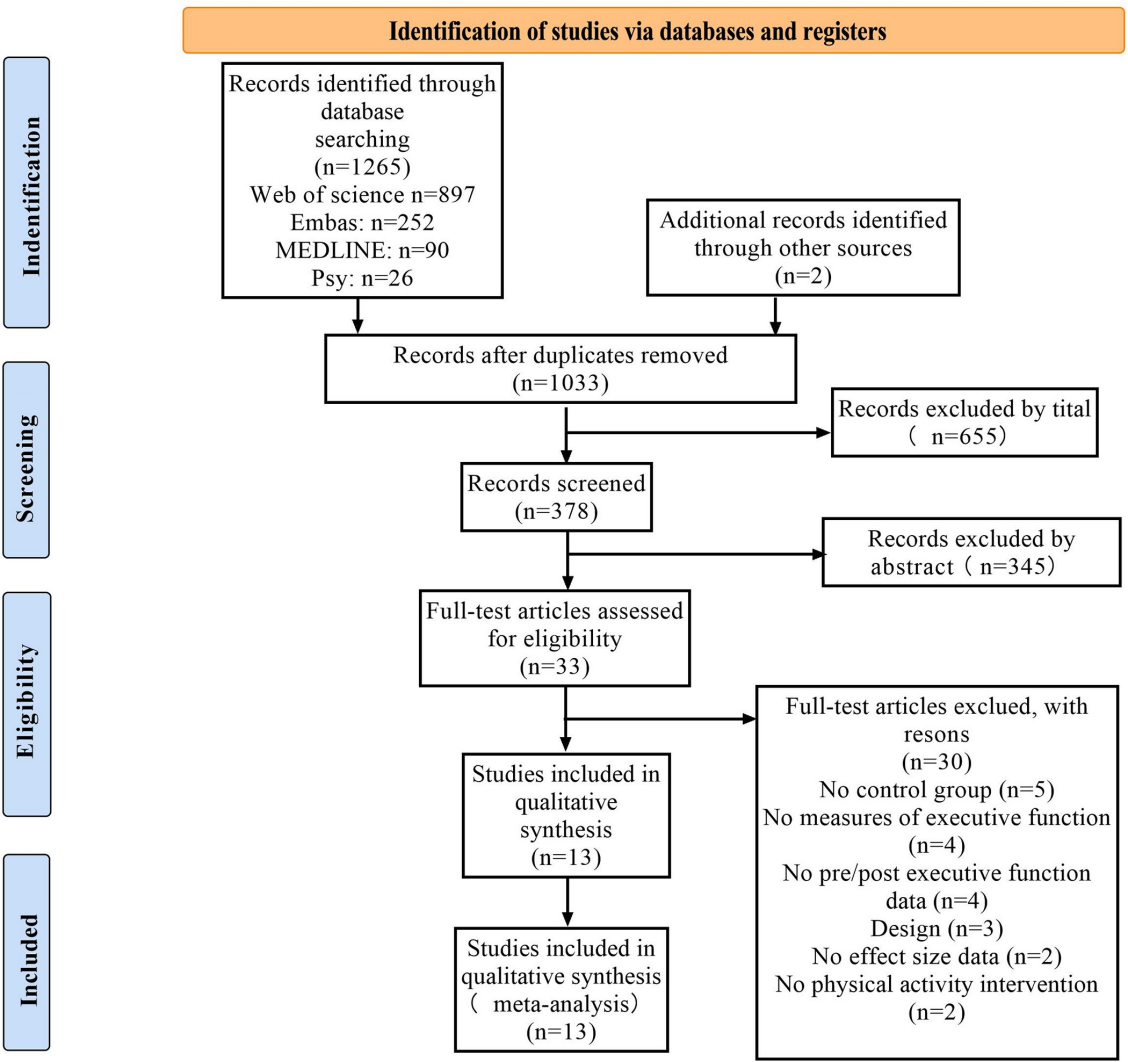
Literature review flowchart.

An initial search of 1267 peer-reviewed articles were conducted, of which two were from published meta-analyses and 1265 were obtained through a search strategy search. Duplicate articles were removed and filtered by title to get 378 articles. After careful examination of these abstracts, 33 articles were deemed eligible for in-depth review. Finally, 20 of these 33 articles were excluded because they did not have a control group, did not test EF, did not have data on EF measured before and after, did not have effect size data, and did not have a physical activity intervention (see Fig 1).

### Characteristics of included studies

To summarize, the meta-analysis included 13 studies, the characteristics of which are detailed in Table 2. The total sample included 752 participants aged from 14 to 75 years. Specifically, the average age of participants in five studies was less than 40 years and in 10 studies the average age was greater than 60 years. In terms of the sub-domains of EF, nine studies evaluated inhibitory control, seven evaluated working memory, and seven evaluated cognitive flexibility. With regards to the geographic location of the included studies, 6 were conducted in the Americas, 6 in Europe, and 1 in Oceania. With regards to the exercise intervention protocols, only 4 studies had interventions lasting longer than 45 minutes. Ten studies had more than three exercise sessions per week, and eight studies had exercise interventions lasting longer than 12 weeks. The primary modifying factors considered in the study were age, session time, exercise frequency, exercise length and sub-domains of EF.

**Table 2:** Studies included for meta-analysis.

Study	Sample (%Males)		Age (Years)		Study region	Grouping	Exercise characteristics	Instrument	EF sub-domains
	Intervention	Control	Intervention	Control					
Albinet et al. 2010 [35]	12 (50%)	12 (58%)	70.9 ± 4.9	70.4 ± 3.4	Europe	Swimming Stretching control	21 weeks, 2 days /week, 60 min/day	Stroop	Inhibitory control
Albinet et al. 2016 [36]	19 (32%)	17 (24%)	67.0 ± 5.0	66.0 ± 5.0	Europe	Aerobic exercise Stretching control	12 weeks, 2 days /week, 60 min/day	Stroop 2-back PMT	Inhibitory control Work memory Flexibility cognitive
Antunes et al. 2015 [37]	23 (NA)	17 (NA)	64.0 ± 3.2	64.8 ± 3.5	USA	Aerobic exercise Stretching control	26 weeks, 3 days /week, 60 min/day	WCST	Inhibitory control
Bliss et al. 2022 [38]	14 (29%)	11 (15%)	67.0 ± 7.0	66.0 ± 9.0	Australia	Aerobic exercise Stretching control	16 weeks, 3 days /week, 45 min/day	Flanker task Spatial Span TMT	Inhibitory control Work memory Flexibility cognitive
Chapman et al.2013 [25]	18 (28%)	19 (26%)	64.0 ± 4.3	64.0 ± 3.6	USA	Aerobic exercise Stretching control	12 weeks, 3 days /week, 40 min/day	TMT	Flexibility cognitive
Edwards et al.2018 [39]	23 (35%)	10 (40%)	21.7 ± 2.8	22.0 ± 2.8	USA	Aerobic exercise Stretching control	7 days /week	Spatial Span	Work memory
Erickson et al.2010. [40]	60 (27%)	60 (40%)	67.6 ± 5.8	65.5 ±5.4	USA	Aerobic exercise Stretching control	52 weeks, 1 day /week, 40 min/day	SMPT	Work memory
Gothe et al. [41]	61 (22%)	57 (25%)	62.1 ± 5.8	62.0 ± 5.4	USA	Yoga Stretching control	8 weeks, 3 days /week	N-Back Task Switching	Work memory Flexibility cognitive
Leckie et al. [26]	47 (32%)	45 (40%)	67.2 ± 5.4	66.4 ± 5.8	USA	Swimming Stretching control	48 weeks, 3 days /week, 40 min/day	Task Switching	Flexibility cognitive
Prehn et al. [42]	11 (36%)	18 (44%)	69.0 ± 5.0	65.0 ± 5.0	Europe	Aerobic exercise Stretching control	24 weeks, 2 days /week, 45 min/day	Stroop DSB TMT	Inhibitory control Work memory Flexibility cognitive
Schwartz et al. [43]	12 (33%)	6 (67%)	35.7 ± 9.6	37.5 ± 12.5	Europe	Sit-to-stand Stretching control	23 weeks	Stroop	Inhibitory control
Torbeyns et al. [44]	21 (38%)	23 (43%)	14.3 ± 0.6	14.3 ± 0.6	Europe	Aerobic exercise Stretching control	22 weeks, 4 days /week, 50 min/day	Stroop	Inhibitory control
Voss et al. [45]	35 (31%)	35 (40%)	65.2 ± 4.4	64.6 ± 4.5	USA	Aerobic exercise Stretching control	52 weeks, 3 days /week, 40 min/day	WCST SMPT Task Switching	Inhibitory control Work memory Flexibility cognitive

WCST, Wisconsin Card Sorting Test; PMT, Plus–Minus Task; TMT, Trail Making Task; SMPT, Spatial Memory Paradigm Task; DSB, Digit span backwards; EF, Executive function.

Results indicate that a significant proportion of the included studies (more than 50%) had high or unclear risks of bias with regards to allocation concealment, blinding of participants and personnel and other biases had low quality (**S2 Fig in S1 Appendix**).

### Overall analysis and sensitivity analysis

**Fig 2:**
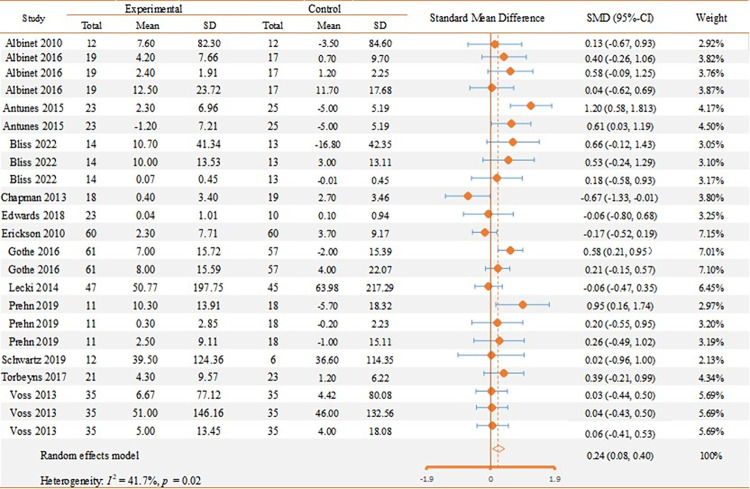
EF overalls forest plot.

As depicted in **Fig 2**, the results of the meta-analysis demonstrate a small but statistically significant positive standardized mean difference in overall EF (SMD = 0.24, 95% CI 0.08 to 0.40) with medium heterogeneity (*I*^2^ = 41.7%, *p*<0.05). The sensitivity analysis revealed that one study was the primary source of elevated heterogeneity [37]. Exclusion of this study resulted in a reduction of heterogeneity (*I*^2^ = 21.8%, *p* = 0.181), with a pooled SMD of 0.17 (95% CI 0.03 to 0.31) for overall EF measures. Results from Egger’s test and a visual assessment of the funnel plot did not provide significant indications of publication bias in the current study (*p* = 0.387). The funnel plot is shown in **S3 Fig in S1 Appendix.**

### Subgroup analysis

Subgroup analyses revealed that relationship between physical activity and EF was significantly moderated by the sub-domains of EF. The results indicated that physical activity had the largest effect on inhibitory control (SMD = 0.38, 95% CI 0.12 to 0.63, *I*^2^ = 23.0%, *p* = 0.238) in sedentary individuals, followed by working memory (SMD = 0.22, 95% CI -0.05 to 0.49, *I*^2^ = 42.8%, *p* = 0.105) and cognitive flexibility (SMD = 0.11, 95% CI -0.18 to 0.41, *I*^2^ = 50.2%, *p* = 0.061) (**S4 Fig in S1 Appendix**). Furthermore, the impact of physical activity on EF was found to be significantly influenced by intervention length, session time, and age, but not by the frequency of exercise. As illustrated by **Table 3**, the SMD for intervention length > 12 weeks was 0.26 (95% CI 0.06 to 0.46) indicating medium heterogeneity (*I*^2^ = 41.4%, *p* = 0.047), whereas the SMD for intervention length ≤ 12 weeks was 0.20 (95% CI -0.08 to 0.47), with medium heterogeneity (*I*^2^ = 43.4%, *p* = 0.089) (**S5 Fig in S1 Appendix**). With respect to the exercise frequency, the SMD for exercise frequency ≥ 3 times/week was 0.24 (95% CI 0.04 to 0.43) with medium heterogeneity (*I*^2^ = 45.0%, *p* = 0.026), and the SMD for < 3 times/week was 0.25 (95% CI -0.05 to 0.52) with medium heterogeneity (*I*^2^ = 31.9%, *p* = 0.184) (**S6 Fig in S1 Appendix**). With respect to the session time, the SMD for session time > 45 min was 0.47 (95% CI 0.22 to 0.77) with small heterogeneity (*I*^2^ = 22.8%, *p* = 0.256), whereas the SMD for ≤ 45 min was 0.17 (95% CI -0.11 to 0.44) with medium heterogeneity (*I*^2^ = 38.0%, *p* = 0.115) (**S7 Fig in S1 Appendix**). With respect to the age, the SMD for participants older than 60 years was 0.25 (95% CI 0.08 to 0.42), with low heterogeneity (*I*^2^ = 0%, *p* = 0.628), whereas the SMD for participants 60 years and younger was 0.17 (95% CI -0.25to 0.59), with medium heterogeneity (*I*^2^ = 46.3%, *p* = 0.012) **(S8 Fig in S1 Appendix)**.

**Table 3:** Moderator analysis of physical activity and EF.

Moderator	Level	n	SMD	95% CI	*I* ^2^	*p*
EF sub-domains	Inhibitory control	9	0.38	0.12 to 0.63	23.0%	0.238
	Working memory	7	0.22	-0.05 to 0.49	42.8%	0.105
	Cognitive flexibility	7	0.11	-0.18 to 0.41	50.2%	0.061
Exercise length	≤ 12 weeks	8	0.20	-0.08. to 0.47	43.4%	0.089
	> 12 weeks	15	0.26	0.06 to 0.46	41.4%	0.047
Exercise frequency	< 3 times/week	7	0.25	-0.05 to 0.52	31.9%	0.184
	≥ 3 times/week	16	0.24	0.04 to 0.43	45.0%	0.026
Session time	> 45 min	7	0.50	0.22 to 0.77	22.8%	0.256
	≤ 45 min	9	0.18	-0.11 to 0.44	38.0%	0.115
Age	≤ 60 years	3	0.18	-0.25 to 0.59	0	0.628
	> 60 years	20	0.25	0.08 to 0.42	46.3%	0.012

## Discussion

The primary purpose of this meta-analysis was to investigate the influence of physical activity on executive function in sedentary individuals. Specifically, this meta-analysis investigated the key moderating variables of physical activity on sedentary individuals, including executive function domain, intervention length, session time, frequency, and age. In the 13 studies included in the meta-analysis, the overall effect revealed a small to moderate benefit of physical activity on executive function in sedentary individuals compared to controls (SMD = 0.24). The current study is the first meta-analysis to explore the effects of a physical activity intervention on executive function in sedentary individuals, with a particular focus on key moderating variables that may influence subsequent cognitive outcomes, such as the EF subdomain and exercise prescription.

### EF Sub‑Domains as a moderator

Although previous studies have shown that physical activity has a favorable influence on EF in sedentary individuals [15]. This meta-analysis offers a valuable contribution to the existing literature by examining the effect of physical activity on sub-domains of EF. The results of the subgroup analysis showed that sedentary individuals who participated in the physical activity showed small to moderate significant improvements in inhibitory control (SMD = 0.38) and working memory (SMD = 0.22), but did not cognitive flexibility (SMD = 0.11). In terms of inhibitory control, our study is consistent with the findings of a recent meta-analysis review investigating the effects of physical activity on inhibitory control in sedentary older adults. A small to moderate positive effect of physical activity on inhibitory control in sedentary individuals was observed [15]. These findings highlight the potential benefits of physical activity in improving inhibitory control in sedentary individuals and emphasize the importance of promoting physical activity in this population. Previous literature suggests that physical activity exhibits a particularly strong association with inhibitory control, as compared to the other two core EFs, in children and adolescents [46,47]. Moreover, our findings also provide evidence that physical activity interventions for sedentary individuals yielded favorable outcomes in terms of enhancing working memory. However, consistent with the results of the present study, previous empirical studies likewise failed to find any effect of physical activity on cognitive flexibility [48]. The results overall suggest that physical activity has a positive effect on two dimensions of EF in sedentary individuals. This supports the potential utility of physical activity interventions as a non-pharmacological approach to improve specific domains of EF in sedentary individuals.

### Exercise prescription variable as a moderator

This meta-analysis review further assessed the important moderating effect of another set of moderating factors (e.g., exercise prescription) on physical activity measures on EF in sedentary individuals. The findings indicated that intervention length is a moderating factor in the effects of EF, with greater benefits associated with longer duration (>12 weeks) physical activity (SMD = 0.26) compared to shorter duration (≤12 weeks) physical activity (SMD = 0.20). This result is similar to the findings of a review by Yan et al. (2022) [49], which highlighted that interventions lasting over 12 weeks were more effective in improving cognitive outcomes in sedentary older adults compared to shorter interventions. These consistent observations reinforce the notion that longer physical activity interventions may be more beneficial for cognitive function in this population. In addition, Colcombe and Kramer (2003) [50] found that studies with intervention durations longer than 6 months had moderate effect sizes that were greater than those for interventions of moderate and short duration. The reason for this may be that prolonged regular physical activity is associated with greater grey matter volume in the prefrontal cortex [51] and increased functional connectivity of the frontal parietal network compared to shorter periods of physical activity [52], which may be associated with greater improvements in EF.

In addition to intervention length, session time was also recognized as moderators in our review. Our results suggest that interventions with >45 minutes of physical activity (SMD = 0.50) have a positive effect on overall EF, while interventions with ≤45 minutes (SMD = 0.17) have no significant effect on EF. This is similar to a previous meta-analysis which showed that >45 minutes of physical activity improved EF [17]. However, the effect of physical activity session time remains controversial. A meta-analysis by Xue et al (2019) [47] showed that physical activity had no significant effect on overall EF when session time was >90 minutes, while physical activity improved overall EF when session time was ≤90 minutes. It should be noted that even participants with a high level of fitness would be anticipated to experience declines in cognitive performance after prolonged physical activity (i.e., 2 h), possibly due to central fatigue caused by heat stress, dehydration, or hypoglycemia [53,54], or with adverse effects on information processing [55]. Interestingly, our review is splitting studies with session time less than 90 minutes into >45 minutes and ≤45 minutes. The duration of physical activity is a key aspect of creating an effective intervention. Our study further extends the understanding of the positive impact of intervention duration and has important implications for future intervention designs targeting sedentary populations.

### Age as a moderator

The results of this review revealed significant differences in improvement of EF after physical activity between individuals with different, and these differences emphasize that physical activity is a beneficial strategy for EF in sedentary older adults (SMD = 0.25). This is consistent with a previous meta-analysis which demonstrated that physical activity can improve EF in sedentary older adults [15]. The reason for this is that normal ageing usually leads to atrophy of the whole brain, changes in functional response of brain, and cognitive ability decline [56]. Therefore, due to their greater cognitive reserve, older people are better able to benefit from physical activity than those in an optimal functional state [20]. Physical activity is associated with larger brain gray matter volumes in brain regions including the hippocampus and prefrontal cortex in older adults [57], and retains white matter integrity, decreased severity of white matter lesions, and improved white matter microstructure [58]. When studies were conducted with children and adolescents, greater benefits were observed among younger participants. Greater benefits were observed in younger participants. This suggests that physical activity may have a stronger and more lasting effect on general cognitive skills during a sensitive period [59]. In addition, previous meta-analyses have found that physical activity has a positive impact on executive function and academic performance in prepubescent children [60]. However, these findings should be interpreted to be cautious, although the current meta-analysis suggests age-related variations in the influence of physical activity on EF in sedentary individuals, there are limited studies with prepubescent children and adolescents to determine. Whether the impact of physical activity is consistent across different developmental stages, including prepubescent children, adolescents, young adults, and older adults, remains to be determined.

### Strengths and limitations

The exclusion of observational studies and the inclusion of only RCT studies is a strength of this meta-analysis. This stringent inclusion criterion enhances the reliability of causal inferences drawn from the study. This systematic review has additional strengths, including the examination of crucial moderators, such as sub-domains of EF, exercise prescription variables, and sample age. Compared to previous reviews, the data in this systematic review allow us to understand the correlation between physical activity and EF in sedentary individuals at a more refined level.

A limitation of the current review was that the included studies had limited control for potential sources of bias and there was no consistent reporting of the extent to which these risks were controlled for, for example, education, cognitive performance at baseline and BMI. Therefore, it is necessary to exercise caution when interpreting the results of the meta-analysis. Another limitation is the insufficient number of studies that include prepubescent children, adolescents and young adults. Therefore, further studies are necessary to establish the effect of physical activity on EF in sedentary individuals of different age groups. Moreover, the third limitation lies in the utilization of gain scores, which overlooks between-group baseline differences. In future research, we intend to employ multilevel models to account for the correlated structure of effects, avoiding mere data averaging. Additionally, we aim to reduce heterogeneity in study outcomes by introducing moderating variables into the model [61]. A fourth limitation is that we did not include exercise type and intensity as moderators. Including them as moderators would have significantly reduced the number of studies that could be included in certain analyses, potentially compromising the robustness of our findings. We prioritized maintaining a more comprehensive data set over introducing these additional moderators. Future research could consider these factors with larger datasets or in more targeted analyses.

## Conclusions

This study is the first meta-analysis to investigate the effects of physical activity on the EF subdomain in sedentary individuals. In addition, we examined how moderating variables, such as exercise prescription variables and age, influenced these relationships. Our findings suggest that physical activity is a potentially effective strategy for improving EF in sedentary individuals, with improvements in inhibitory control and working memory. Based on the findings of this review, it is recommended that future intervention sessions should not exceed 45 minutes, and the exercise length needs to last more than 12 weeks, as this has been shown to have the greatest benefit for executive function in sedentary individuals. Whether the frequency of exercise was 1–2 or 3–4 times per week, physical activity had a positive effect on executive function and there was no significant difference between the two frequencies. Sedentary older individuals appeared to benefit more from physical activity. These results may be used as the foundation for suggestions on how to encourage sedentary people to engage in physical exercise on a regular basis. These could consequently be interesting to healthcare providers.

## Supporting information

S1 Checklist PRISMA 2020 checklist. (PDF)

S1 Appendix Extended results on physical activity to improve executive function in sedentary individuals. It includes a search strategy for literature organization, an assessment framework for evaluating the quality of the literature (S1 and S2 Figs), funnel plots (S3 Fig) and subgroup forest plots for assessing publication bias (S4-S8 Figs), and PRISMA Checklist.
(DOCX)
