# Supplementary material for: Physical activity on executive function in sedentary individuals: Systematic review and meta-analysis of randomized controlled trials
Source: PLoS One. 2023 Dec 7;18(12):e0294251. doi: 10.1371/journal.pone.0294251 (PMC10703253; doi:10.1371/journal.pone.0294251)
Supplement: S1 Appendix — It includes a search strategy for literature organization, an assessment framework for evaluating the quality of the literature (S1 and S2 Figs), funnel plots (S3 Fig) and subgroup forest plots for assessing publication bias (S4-S8 Figs), and PRISMA Checklist. (DOCX) [file pone.0294251.s002.DOCX]

**S1 Appendix**

**Physical activity interventions on executive function in sedentary individuals: a systematic review with meta-analysis**

Contents

[Search strategies 1](#_Toc150119883)

[PubMed 1](#_Toc150119884)

[Embase 3](#_Toc150119885)

[PsycINFO 5](#_Toc150119886)

[Web of Science 6](#_Toc150119887)

[Literature quality assessment 7](#_Toc150119888)

[Funnel plot 9](#_Toc150119889)

[Subgroup forest plot 10](#_Toc150119890)

[PRISMA 2020 Checklist 15](#_Toc150119891)

[List of literature included in the Meta-analysis 19](#_Toc150119893)

# Search strategies

## PubMed

#1 "Executive Function"[Mesh]

#2 ((Executive Function[Title/Abstract]) OR (Function, Executive[Title/Abstract])) OR (Functions, Executive[Title/Abstract]) OR (Executive Control[Title/Abstract]) OR (Executive Controls [Title/Abstract])

#3 ((((((((((((Executive Functions[Mesh]) OR (Executive Control[Title/Abstract])) OR (working memory[Title/Abstract])) OR (inhibitory control[Title/Abstract])) OR (cognitive flexibility[Title/Abstract])) OR (cognition[Title/Abstract])) OR (cognitive function[Title/Abstract])) OR (cognitive performance[Title/Abstract])) OR (inhibition[Title/Abstract])) OR (update[Title/Abstract])) OR (switch[Title/Abstract]))

#4 "Exercise"[Mesh]

#5 (((((((((((((((((((Physical Activity[Title/Abstract]) OR (Activities, Physical[Title/Abstract])) OR (Activity, Physical[Title/Abstract])) OR (Physical Activities[Title/Abstract])) OR (Exercise, Physical[Title/Abstract])) OR (Exercises, Physical[Title/Abstract])) OR (Physical Exercise[Title/Abstract])) OR (Physical Exercises[Title/Abstract])) OR (Exercise, Isometric[Title/Abstract])) OR (Exercises, Isometric[Title/Abstract])) OR (Isometric Exercises[Title/Abstract])) OR (Isometric Exercise[Title/Abstract])) OR (Exercise, Aerobic[Title/Abstract])) OR (Aerobic Exercise[Title/Abstract])) OR (Aerobic Exercises[Title/Abstract])) OR (Exercises, Aerobic[Title/Abstract])) OR (Exercise Training[Title/Abstract])) OR (Exercise Trainings[Title/Abstract])) OR (Training, Exercise[Title/Abstract])) OR (Trainings, Exercise[Title/Abstract])

#6 ("Exercise"[Mesh]) OR ((((((((((((((((((((Physical Activity[Title/Abstract]) OR (Activities, Physical[Title/Abstract])) OR (Activity, Physical[Title/Abstract])) OR (Physical Activities[Title/Abstract])) OR (Exercise, Physical[Title/Abstract])) OR (Exercises, Physical[Title/Abstract])) OR (Physical Exercise[Title/Abstract])) OR (Physical Exercises[Title/Abstract])) OR (Exercise, Isometric[Title/Abstract])) OR (Exercises, Isometric[Title/Abstract])) OR (Isometric Exercises[Title/Abstract])) OR (Isometric Exercise[Title/Abstract])) OR (Exercise, Aerobic[Title/Abstract])) OR (Aerobic Exercise[Title/Abstract])) OR (Aerobic Exercises[Title/Abstract])) OR (Exercises, Aerobic[Title/Abstract])) OR (Exercise Training[Title/Abstract])) OR (Exercise Trainings[Title/Abstract])) OR (Training, Exercise[Title/Abstract])) OR (Trainings, Exercise[Title/Abstract]))

#7 "Sedentary Behavior"[Mesh]

#8 (((((((((Behavior, Sedentary[Title/Abstract]) OR (Sedentary Behaviors[Title/Abstract])) OR (Sedentary Lifestyle[Title/Abstract])) OR (Lifestyle, Sedentary[Title/Abstract])) OR (Physical Inactivity[Title/Abstract])) OR (Inactivity, Physical[Title/Abstract])) OR (Lack of Physical Activity[Title/Abstract])) OR (Sedentary Time[Title/Abstract])) OR (Sedentary Times[Title/Abstract])) OR (Time, Sedentary[Title/Abstract])

#9 ("Sedentary Behavior"[Mesh]) OR ((((((((((Behavior, Sedentary[Title/Abstract]) OR (Sedentary Behaviors[Title/Abstract])) OR (Sedentary Lifestyle[Title/Abstract])) OR (Lifestyle, Sedentary[Title/Abstract])) OR (Physical Inactivity[Title/Abstract])) OR (Inactivity, Physical[Title/Abstract])) OR (Lack of Physical Activity[Title/Abstract])) OR (Sedentary Time[Title/Abstract])) OR (Sedentary Times[Title/Abstract])) OR (Time, Sedentary[Title/Abstract]))

#10 randomized controlled trial[Publication Type] OR randomized[Title/Abstract] OR placebo[Title/Abstract]

#11 ((((((((((((Executive Function[Mesh]) OR (Executive Control[Title/Abstract])) OR (working memory[Title/Abstract])) OR (inhibitory control[Title/Abstract])) OR (cognitive flexibility[Title/Abstract])) OR (cognition[Title/Abstract])) OR (cognitive function[Title/Abstract])) OR (cognitive performance[Title/Abstract])) OR (inhibition[Title/Abstract])) OR (update[Title/Abstract])) OR (switch[Title/Abstract])) AND (("Exercise"[Mesh]) OR ((((((((((((((((((((Physical Activity[Title/Abstract]) OR (Activities, Physical[Title/Abstract])) OR (Activity, Physical[Title/Abstract])) OR (Physical Activities[Title/Abstract])) OR (Exercise, Physical[Title/Abstract])) OR (Exercises, Physical[Title/Abstract])) OR (Physical Exercise[Title/Abstract])) OR (Physical Exercises[Title/Abstract])) OR (Exercise, Isometric[Title/Abstract])) OR (Exercises, Isometric[Title/Abstract])) OR (Isometric Exercises[Title/Abstract])) OR (Isometric Exercise[Title/Abstract])) OR (Exercise, Aerobic[Title/Abstract])) OR (Aerobic Exercise[Title/Abstract])) OR (Aerobic Exercises[Title/Abstract])) OR (Exercises, Aerobic[Title/Abstract])) OR (Exercise Training[Title/Abstract])) OR (Exercise Trainings[Title/Abstract])) OR (Training, Exercise[Title/Abstract])) OR (Trainings, Exercise[Title/Abstract]))))

#12 ((((((((((((Executive Function[Mesh]) OR (Executive Control[Title/Abstract])) OR (working memory[Title/Abstract])) OR (inhibitory control[Title/Abstract])) OR (cognitive flexibility[Title/Abstract])) OR (cognition[Title/Abstract])) OR (cognitive function[Title/Abstract])) OR (cognitive performance[Title/Abstract])) OR (inhibition[Title/Abstract])) OR (update[Title/Abstract])) OR (switch[Title/Abstract])) AND (("Exercise"[Mesh]) OR ((((((((((((((((((((Physical Activity[Title/Abstract]) OR (Activities, Physical[Title/Abstract])) OR (Activity, Physical[Title/Abstract])) OR (Physical Activities[Title/Abstract])) OR (Exercise, Physical[Title/Abstract])) OR (Exercises, Physical[Title/Abstract])) OR (Physical Exercise[Title/Abstract])) OR (Physical Exercises[Title/Abstract])) OR (Exercise, Isometric[Title/Abstract])) OR (Exercises, Isometric[Title/Abstract])) OR (Isometric Exercises[Title/Abstract])) OR (Isometric Exercise[Title/Abstract])) OR (Exercise, Aerobic[Title/Abstract])) OR (Aerobic Exercise[Title/Abstract])) OR (Aerobic Exercises[Title/Abstract])) OR (Exercises, Aerobic[Title/Abstract])) OR (Exercise Training[Title/Abstract])) OR (Exercise Trainings[Title/Abstract])) OR (Training, Exercise[Title/Abstract])) OR (Trainings, Exercise[Title/Abstract])))) AND (randomized controlled trial[Publication Type] OR randomized[Title/Abstract] OR placebo[Title/Abstract]))

#13 (((((((((((((((((((((Physical Activity[Title/Abstract]) OR (Activities, Physical[Title/Abstract])) OR (Activity, Physical[Title/Abstract])) OR (Physical Activities[Title/Abstract])) OR (Exercise, Physical[Title/Abstract])) OR (Exercises, Physical[Title/Abstract])) OR (Physical Exercise[Title/Abstract])) OR (Physical Exercises[Title/Abstract])) OR (Exercise, Isometric[Title/Abstract])) OR (Exercises, Isometric[Title/Abstract])) OR (Isometric Exercises[Title/Abstract])) OR (Isometric Exercise[Title/Abstract])) OR (Exercise, Aerobic[Title/Abstract])) OR (Aerobic Exercise[Title/Abstract])) OR (Aerobic Exercises[Title/Abstract])) OR (Exercises, Aerobic[Title/Abstract])) OR (Exercise Training[Title/Abstract])) OR (Exercise Trainings[Title/Abstract])) OR (Training, Exercise[Title/Abstract])) OR (Trainings, Exercise[Title/Abstract]))) AND (((((((((((((Executive Function[Title/Abstract]) OR (Executive Control[Title/Abstract])) OR (working memory[Title/Abstract])) OR (inhibitory control[Title/Abstract])) OR (cognitive flexibility[Title/Abstract])) OR (cognition[Title/Abstract])) OR (cognitive function[Title/Abstract])) OR (cognitive performance[Title/Abstract])) OR (inhibition[Title/Abstract])) OR (update[Title/Abstract])) OR (switch[Title/Abstract])) AND (((((randomized controlled trial[Title/Abstract]) OR (randomized[Title/Abstract])) OR (placebo[Title/Abstract])) OR (control clinical trial[Title/Abstract])) OR (randomized clinical trial[Title/Abstract]))) AND (((((((((((Behavior, Sedentary[Title/Abstract]) OR (Sedentary Behavior[Title/Abstract])) OR (Sedentary Behaviors[Title/Abstract])) OR (Sedentary Lifestyle[Title/Abstract])) OR (Lifestyle, Sedentary[Title/Abstract])) OR (Physical Inactivity[Title/Abstract])) OR (Inactivity, Physical[Title/Abstract])) OR (Lack of Physical Activity[Title/Abstract])) OR (Sedentary Time[Title/Abstract])) OR (Sedentary Times[Title/Abstract])) OR (Time, Sedentary[Title/Abstract])))

## Embase

#1 'executive function'

#2 'executive functions':ab,ti OR ' function, executive ':ab,ti OR 'executive control ':ab,ti OR 'executive Controls':ab,ti

#3 'executive function' OR (executive functions':ab,ti OR 'function, executive ':ab,ti OR 'executive control ':ab,ti OR 'executive controls':ab,ti OR 'working memory':ab,ti OR 'inhibitory control':ab,ti OR 'cognitive flexibility':ab,ti OR 'cognition':ab,ti OR 'cognitive function':ab,ti OR 'cognitive performance':ab,ti OR 'inhibition':ab,ti OR 'update':ab,ti OR 'switch':ab,ti)

#4 'exercises'

#5 'physical activity':ab,ti OR 'activities, physical':ab,ti OR 'activity, physical':ab,ti OR 'physical activities':ab,ti OR 'exercise, physical':ab,ti OR 'exercises, physical':ab,ti OR 'physical exercise':ab,ti OR 'physical exercises':ab,ti OR 'exercise, isometric':ab,ti OR 'exercises, isometric':ab,ti OR 'isometric exercises':ab,ti OR 'isometric exercise':ab,ti OR 'exercise aerobic':ab,ti OR 'aerobic exercise':ab,ti OR 'aerobic exercises':ab,ti OR 'exercises, aerobic':ab,ti OR 'exercise training':ab,ti OR 'exercise trainings':ab,ti OR 'training, exercise':ab,ti OR 'trainings, exercise':ab,ti

#6 'exercises' OR ('physical activity':ab,ti OR 'activities, physical':ab,ti OR 'activity, physical':ab,ti OR 'physical activities':ab,ti OR 'exercise, physical':ab,ti OR 'exercises, physical':ab,ti OR 'physical exercise':ab,ti OR 'physical exercises':ab,ti OR 'exercise, isometric':ab,ti OR 'exercises, isometric':ab,ti OR 'isometric exercises':ab,ti OR 'isometric exercise':ab,ti OR 'exercise aerobic':ab,ti OR 'aerobic exercise':ab,ti OR 'aerobic exercises':ab,ti OR 'exercises, aerobic':ab,ti OR 'exercise training':ab,ti OR 'exercise trainings':ab,ti OR 'training, exercise':ab,ti OR 'trainings, exercise':ab,ti)

#7 'sedentary behavior'/exp

#8 'behavior, sedentary':ab,ti OR 'sedentary behaviors':ab,ti OR 'sedentary lifestyle':ab,ti OR 'lifestyle, sedentary':ab,ti OR 'physical inactivity':ab,ti OR 'inactivity, physical':ab,ti OR 'lack of physical activity':ab,ti OR 'sedentary time':ab,ti OR 'sedentary times':ab,ti OR 'time, sedentary':ab,ti

#9 'sedentary behavior'/exp OR ('behavior, sedentary':ab,ti OR 'sedentary behaviors':ab,ti OR 'sedentary lifestyle':ab,ti OR 'lifestyle, sedentary':ab,ti OR 'physical inactivity':ab,ti OR 'inactivity, physical':ab,ti OR 'lack of physical activity':ab,ti OR 'sedentary time':ab,ti OR 'sedentary times':ab,ti OR 'time, sedentary':ab,ti)

#10 'randomized controlled trial':ab,ti OR 'randomized':ab,ti OR 'placebo':ab,ti

#12 'executive function' OR (executive functions':ab,ti OR 'function, executive ':ab,ti OR 'executive control ':ab,ti OR 'executive controls':ab,ti OR 'working memory':ab,ti OR 'inhibitory control':ab,ti OR 'cognitive flexibility':ab,ti OR 'cognition':ab,ti OR 'cognitive function':ab,ti OR 'cognitive performance':ab,ti OR 'inhibition':ab,ti OR 'update':ab,ti OR 'switch':ab,ti) AND ('exercises' OR ('physical activity':ab,ti OR 'activities, physical':ab,ti OR 'activity, physical':ab,ti OR 'physical activities':ab,ti OR 'exercise, physical':ab,ti OR 'exercises, physical':ab,ti OR 'physical exercise':ab,ti OR 'physical exercises':ab,ti OR 'exercise, isometric':ab,ti OR 'exercises, isometric':ab,ti OR 'isometric exercises':ab,ti OR 'isometric exercise':ab,ti OR 'exercise aerobic':ab,ti OR 'aerobic exercise':ab,ti OR 'aerobic exercises':ab,ti OR 'exercises, aerobic':ab,ti OR 'exercise training':ab,ti OR 'exercise trainings':ab,ti OR 'training, exercise':ab,ti OR 'trainings, exercise':ab,ti)) AND ('randomized controlled trial':ab,ti OR 'randomized':ab,ti OR 'placebo':ab,ti)

#13 'executive function' OR (executive functions':ab,ti OR 'function, executive ':ab,ti OR 'executive control ':ab,ti OR 'executive controls':ab,ti OR 'working memory':ab,ti OR 'inhibitory control':ab,ti OR 'cognitive flexibility':ab,ti OR 'cognition':ab,ti OR 'cognitive function':ab,ti OR 'cognitive performance':ab,ti OR 'inhibition':ab,ti OR 'update':ab,ti OR 'switch':ab,ti) AND ('sedentary behavior'/exp OR ('behavior, sedentary':ab,ti OR 'sedentary behaviors':ab,ti OR 'sedentary lifestyle':ab,ti OR 'lifestyle, sedentary':ab,ti OR 'physical inactivity':ab,ti OR 'inactivity, physical':ab,ti OR 'lack of physical activity':ab,ti OR 'sedentary time':ab,ti OR 'sedentary times':ab,ti OR 'time, sedentary':ab,ti)) AND ('cohort studies'/exp OR 'longitudinal studies'/exp OR 'follow-up studies'/exp OR 'prospective studies'/exp OR 'retrospective studies'/exp OR 'cohort' OR 'longitudinal'/exp OR 'prospective' OR 'retrospective') AND ('randomized controlled trial':ab,ti OR 'randomized':ab,ti OR 'placebo':ab,ti) AND ('exercises' OR ('physical activity':ab,ti OR 'activities, physical':ab,ti OR 'activity, physical':ab,ti OR 'physical activities':ab,ti OR 'exercise, physical':ab,ti OR 'exercises, physical':ab,ti OR 'physical exercise':ab,ti OR 'physical exercises':ab,ti OR 'exercise, isometric':ab,ti OR 'exercises, isometric':ab,ti OR 'isometric exercises':ab,ti OR 'isometric exercise':ab,ti OR 'exercise aerobic':ab,ti OR 'aerobic exercise':ab,ti OR 'aerobic exercises':ab,ti OR 'exercises, aerobic':ab,ti OR 'exercise training':ab,ti OR 'exercise trainings':ab,ti OR 'training, exercise':ab,ti OR 'trainings, exercise':ab,ti))

## PsycINFO

#1 TI executive function OR TI executive functions OR TI function, execution OR TI executive control OR TI executive controls OR TI inhibitory control OR TI working memory OR TI cognitive flexibility OR TI inhibition OR TI cognition OR TI cognitive function OR TI switch OR TI update OR TI cognitive performance

#2 TI Exercises OR TI Physical Activity OR TI Activities, Physical OR TI Activity, Physical OR TI Physical Activities OR TI Exercise, Physical OR TI Exercises, Physical OR TI Physical Exercise OR TI Physical Exercises OR TI Exercise, Isometric OR TI Exercises, Isometric OR TI Isometric Exercises OR TI Isometric Exercise OR TI Exercise, Aerobic OR TI Aerobic Exercise OR TI Aerobic Exercises OR TI Exercises, Aerobic OR TI Exercise Training OR TI Exercise Trainings OR TI Training, Exercise OR TI Trainings, Exercise

#3 TI Sedentary Behavior OR TI Behavior, Sedentary OR TI Sedentary Behaviors OR TI Sedentary Lifestyle OR TI Lifestyle, Sedentary OR TI Physical Inactivity OR TI Inactivity, Physical OR TI Lack of Physical Activity OR TI Sedentary Time OR TI Sedentary Times OR TI Time, Sedentary

#4 ( TI Exercises OR TI Physical Activity OR TI Activities, Physical OR TI Activity, Physical OR TI Physical Activities OR TI Exercise, Physical OR TI Exercises, Physical OR TI Physical Exercise OR TI Physical Exercises OR TI Exercise, Isometric OR TI Exercises, Isometric OR TI Isometric Exercises OR TI Isometric Exercise OR TI Exercise, Aerobic OR TI Aerobic Exercise OR TI Aerobic Exercises OR TI Exercises, Aerobic OR TI Exercise Training OR TI Exercise Trainings OR TI Training, Exercise OR TI Trainings, Exercise ) AND ( TI executive function OR TI executive functions OR TI function, execution OR TI executive control OR TI executive controls OR TI inhibitory control OR TI working memory OR TI cognitive flexibility OR TI inhibition OR TI cognition OR TI cognitive function OR TI switch OR TI update OR TI cognitive performance ) AND ( TI Sedentary Behavior OR TI Behavior, Sedentary OR TI Sedentary Behaviors OR TI Sedentary Lifestyle OR TI Lifestyle, Sedentary OR TI Physical Inactivity OR TI Inactivity, Physical OR TI Lack of Physical Activity OR TI Sedentary Time OR TI Sedentary Times OR TI Time, Sedentary )

## Web of Science

#1 TS=(Executive Function OR Executive Control OR executive functions OR function, execution OR Executive Control OR working memory OR inhibitory control OR cognitive flexibility OR cognition OR cognitive function OR cognitive performance OR inhibition OR update OR switch)

#2 TS=(Exercises, OR Physical Activity, OR Activities, Physical, OR Activity, Physical, OR Physical Activities, OR Exercise, Physical, OR Exercises, Physical, OR Physical Exercise, OR Physical Exercises, OR Exercise, Isometric, OR Exercises, Isometric, OR Isometric Exercises, OR Isometric Exercise, OR Exercise, Aerobic, OR Aerobic Exercise, OR Aerobic Exercises, OR Exercises, Aerobic, OR Exercise Training, OR Exercise Trainings, OR Training, Exercise, OR Trainings, Exercise)

#3 TS=(Sedentary Behavior, OR Behavior, Sedentary, OR Sedentary Behaviors, OR Sedentary Lifestyle, OR Lifestyle, Sedentary, OR Physical Inactivity, OR Inactivity, Physical, OR Lack of Physical Activity, OR Sedentary Time, OR Sedentary Times, OR Time, Sedentary)

#4 TS=(randomized controlled trial OR randomized OR placebo)

#6 #1AND#2AND#3AND#4AND

# Literature quality assessment


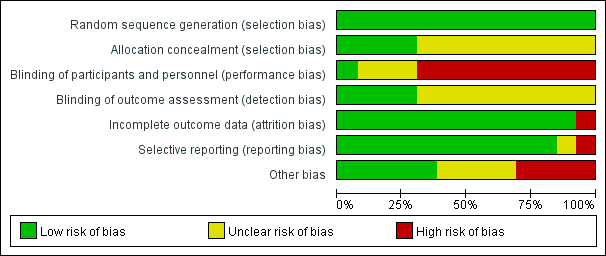


Figure S1. Risk of bias for summary quality. Green, the risk of bias was low; Yellow, the risk of bias was unclear; Red, the risk of bias was high.


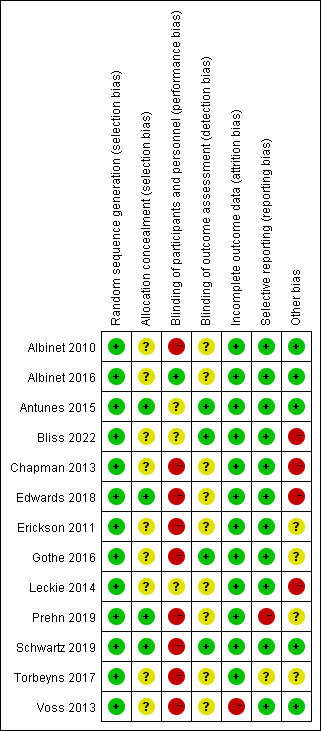


Figure S2. Assessment for the risk of bias in the included studies. Green circle, the risk of bias was low; Yellow circle, the risk of bias was unclear; Red circle, the risk of bias was high.

# Funnel plot


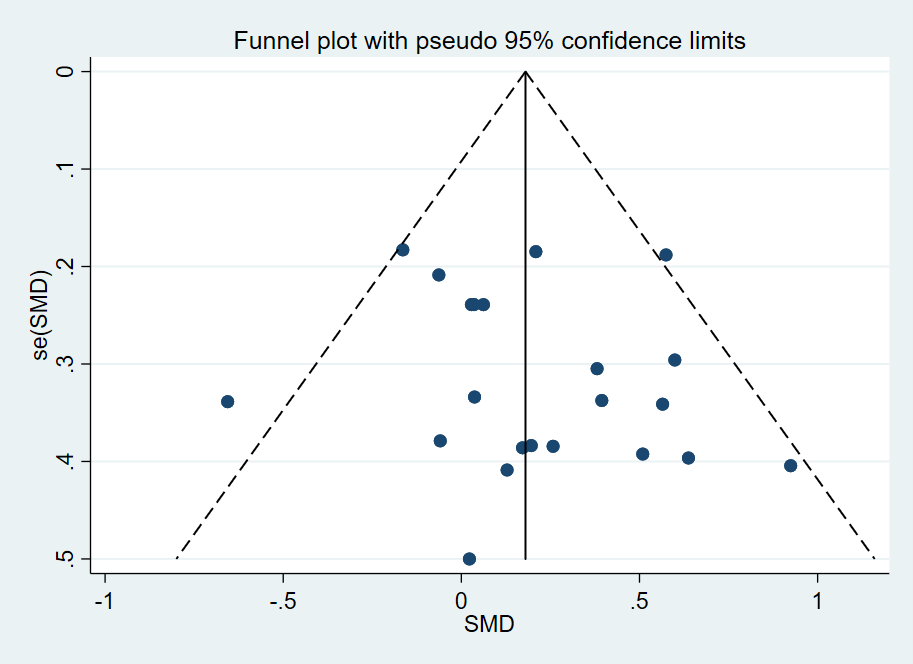


Figure S3. EF overalls funnel plot.

# Subgroup forest plot


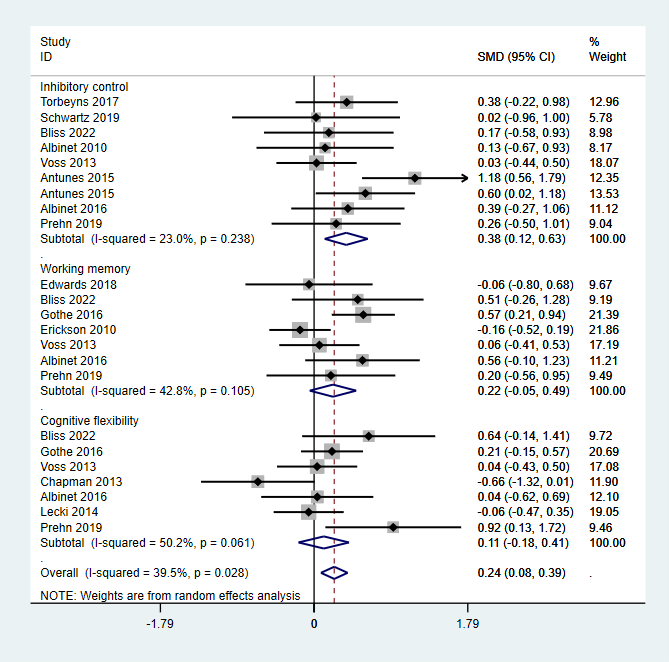


Figure S4. EF sub-domains subgroup forest plot.


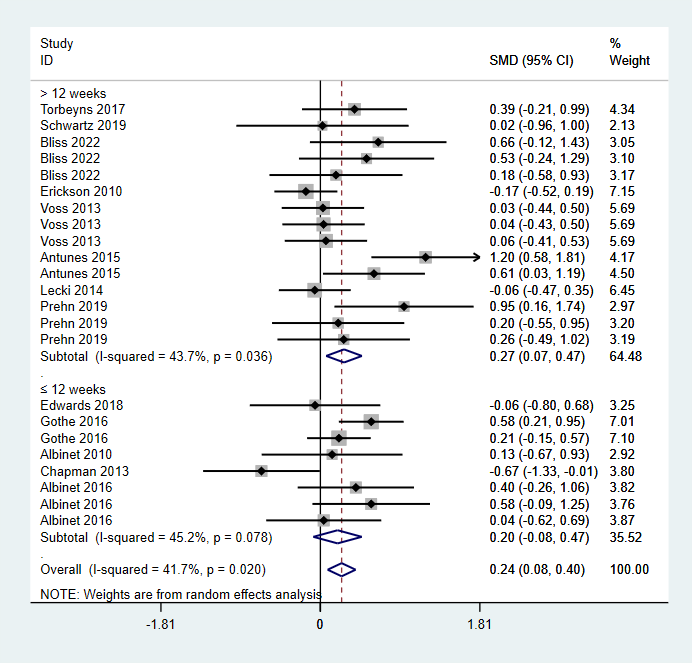


Figure S5. Exercise length subgroup forest plot.


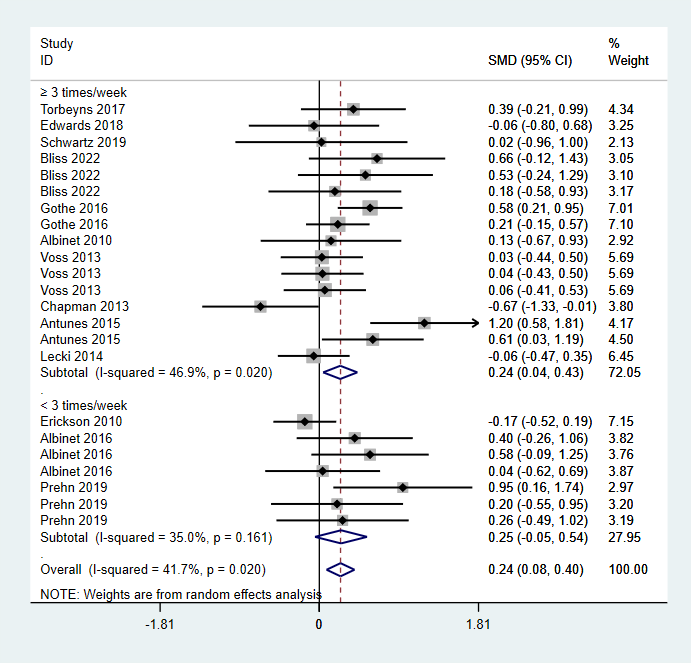


Figure S6. Exercise frequency subgroup forest plot.


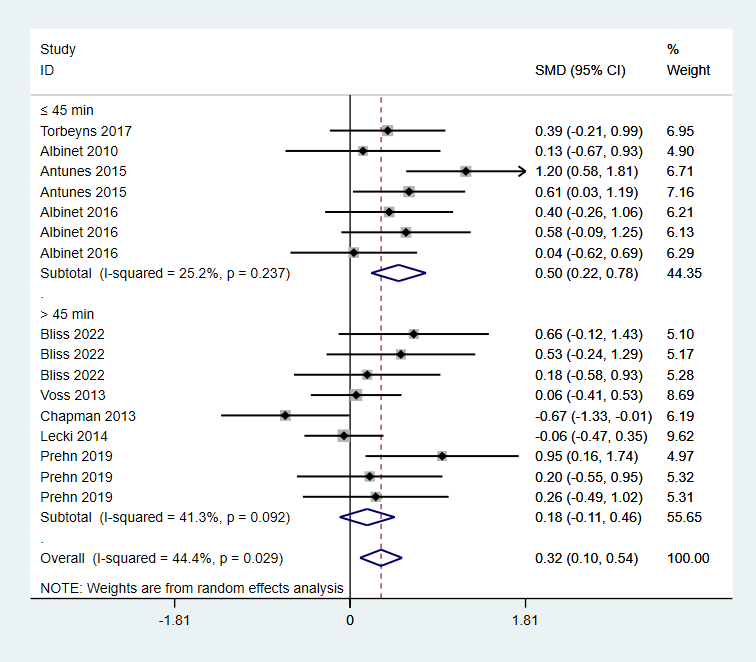


Figure S7. Session time subgroup forest plot.


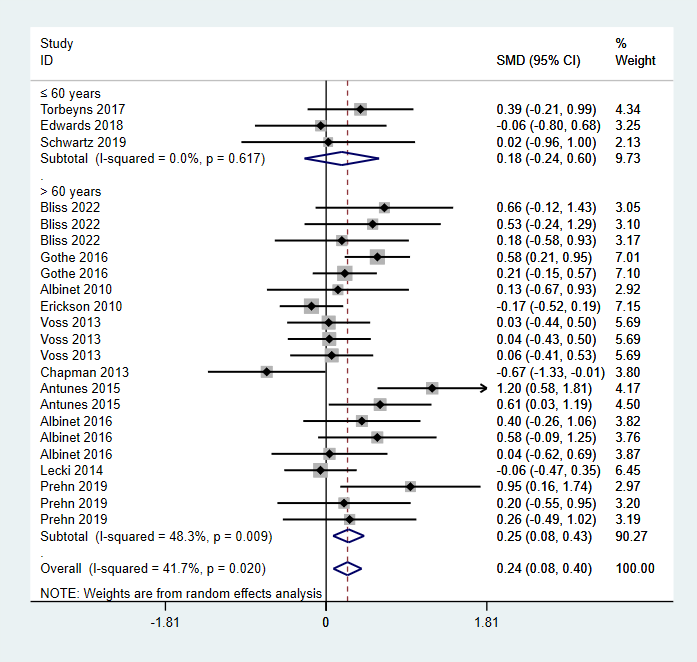


Figure S8. Age subgroup forest plot.

# PRISMA 2020 Checklist

| **Section/topic** | **Item No** | **Checklist item** | **Reported on Page Number/Line Number** | **Reported on Section/Paragraph** |
| --- | --- | --- | --- | --- |
| **TITLE** | | | | |
| Title | 1 | Identify the report as a systematic review. | Page1/line2-2 | Title page |
| **ABSTRACT** | | | | |
| Abstract | 2 | See the PRISMA 2020 for Abstracts checklist (Table 2). | Page1/line11-34 | Abstract |
| **INTRODUCTION** | | | | |
| Rationale | 3 | Describe the rationale for the review in the context of existing knowledge. | Page2/line38-47 | Introduction/Paragraph1 |
| Objectives | 4 | Provide an explicit statement of the objective(s) or question(s) the review addresses. | Page2/line85-88 | Introduction/Paragraph5 |
| **METHODS** | | | | |
| Eligibility criteria | 5 | Specify the inclusion and exclusion criteria for the review and how studies were grouped for the syntheses. | Page3/line117-129 | Methods/Paragraph3 |
| Information sources | 6 | Specify all databases, registers, websites, organisations, reference lists and other sources searched or consulted to identify studies. Specify the date when each source was last searched or consulted. | Page3/line98-115 | Methods/Paragraph2 |
| Search strategy | 7 | Present the full search strategies for all databases, registers and websites, including any filters and limits used. | Page3/line98-113 | Methods/Paragraph2 |
| Selection process | 8 | Specify the methods used to decide whether a study met the inclusion criteria of the review, including how many reviewers screened each record and each report retrieved, whether they worked independently, and if applicable, details of automation tools used in the process. | Page3/line98-103 | Methods/Paragraph2 |
| Data collection process | 9 | Specify the methods used to collect data from reports, including how many reviewers collected data from each report, whether they worked independently, any processes for obtaining or confirming data from study investigators, and if applicable, details of automation tools used in the process. | Page3/line98-116 | Methods/Paragraph2 |
| Data items | 10a | List and define all outcomes for which data were sought. Specify whether all results that were compatible with each outcome domain in each study were sought (e.g. for all measures, time points, analyses), and if not, the methods used to decide which results to collect. | Page3/line131-136 | Methods/Paragraph4 |
|  | 10b | List and define all other variables for which data were sought (e.g. participant and intervention characteristics, funding sources). Describe any assumptions made about any missing or unclear information. | Page3/line132-136 | Methods/Paragraph4 |

| Study risk of bias assessment | 11 | Specify the methods used to assess risk of bias in the included studies, including details of the tool(s) used, how many reviewers assessed each study and whether they worked independently, and if applicable, details of automation tools used in the process. | Page3-4/line138-147 | Methods/Paragraph5 |
| --- | --- | --- | --- | --- |
| Effect measures | 12 | Specify for each outcome the effect measure(s) (e.g. risk ratio, mean difference) used in the synthesis or presentation of results. | Page4/line149-153 | Methods/Paragraph6 |
| Synthesis methods | 13a | Describe the processes used to decide which studies were eligible for each synthesis. | Page4/line149-153 | Methods/Paragraph6 |
|  | 13b | Describe any methods required to prepare the data for presentation or synthesis, such as handling of missing summary statistics, or data conversions. | Page4/line155-157 | Methods/Paragraph6 |
|  | 13c | Describe any methods used to tabulate or visually display results of individual studies and syntheses. | Page4/line157-160 | Methods/Paragraph6 |
|  | 13d | Describe any methods used to synthesize results and provide a rationale for the choice(s). If meta-analysis was performed, describe the model(s), method(s) to identify the presence and extent of statistical heterogeneity, and software package(s) used. | Page4/line153-154, 167-168 | Methods/Paragraph6-7 |
|  | 13e | Describe any methods used to explore possible causes of heterogeneity among study results. | Page4/line154-155 | Methods/Paragraph6 |
|  | 13f | Describe any sensitivity analyses conducted to assess robustness of the synthesized results. | Page44/line156-157 | Methods/Paragraph6 |
| Reporting bias assessment | 14 | Describe any methods used to assess risk of bias due to missing results in a synthesis (arising from reporting biases). | Page4/line158-160 | Methods/Paragraph6 |
| Certainty assessment | 15 | Describe any methods used to assess certainty (or confidence) in the body of evidence for an outcome. | Page4/line162-167 | Methods/Paragraph7 |
| **RESULTS** | | | | |
| Study selection | 16a | Describe the results of the search and selection process, from the number of records identified in the search to the number of studies included in the review, ideally using a flow diagram. | Page4/line170-175 | Results/Paragraph1 |
|  | 16b | Cite studies that met many but not all inclusion criteria (‘near-misses’) and explain why they were excluded. | Page4/line172-175 | Results/Paragraph1 |
| Study characteristics | 17 | Cite each included study and present its characteristics. | Page4/line177-187 | Results/Paragraph2 |
| Risk of bias in studies | 18 | Present assessments of risk of bias for each included study. | Page4/line188-191 | Results/Paragraph3 |
| Results of individual studies | 19 | For all outcomes, present, for each study: (a) summary statistics for each group (where appropriate) and (b) an effect estimate and its precision (e.g. confidence/credible interval), ideally using structured tables or plots. | Page4-5/line192-216 | Results/Paragraph4-5 |

| Results of syntheses | 20a | For each synthesis, briefly summarise the characteristics and risk of bias among contributing studies. | Page4-5/line192-216 | Results/Paragraph4-5 |
| --- | --- | --- | --- | --- |
|  | 20b | Present results of all statistical syntheses conducted. If meta-analysis was done, present for each the summary estimate and its precision (e.g. confidence/credible interval) and measures of statistical heterogeneity. If comparing groups, describe the direction of the effect. | Page4-5/line192-216 | Results/Paragraph4-5 |
|  | 20c | Present results of all investigations of possible causes of heterogeneity among study results. | Page5/line195-196 | Results/Paragraph4 |
|  | 20d | Present results of all sensitivity analyses conducted to assess the robustness of the synthesized results. | Page5/line196-199 | Results/Paragraph4 |
| Reporting biases | 21 | Present assessments of risk of bias due to missing results (arising from reporting biases) for each synthesis assessed. | Page5/line196-199 | Results/Paragraph4 |
| Certainty of evidence | 22 | Present assessments of certainty (or confidence) in the body of evidence for each outcome assessed. | Page5/line201-216 | Results/Paragraph5 |
| **DISCUSSION** | | | | |
| Discussion | 23a | Provide a general interpretation of the results in the context of other evidence. | Page8/line226-233 | Discussion/Paragraph1 |
|  | 23b | Discuss any limitations of the evidence included in the review. | Page9/line304-310 | Discussion/Paragraph7 |
|  | 23c | Discuss any limitations of the review processes used. | Page9/line304-310 | Discussion/Paragraph7 |
|  | 23d | Discuss implications of the results for practice, policy, and future research. | Page9/line312-321 | Discussion/Paragraph8 |
| **OTHER INFORMATION** | | | | |
| Registration and protocol | 24a | Provide registration information for the review, including register name and registration number, or state that the review was not registered. | NA | NA |
|  | 24b | Indicate where the review protocol can be accessed, or state that a protocol was not prepared. | NA | NA |
|  | 24c | Describe and explain any amendments to information provided at registration or in the protocol. | NA | NA |
| Support | 25 | Describe sources of financial or non-financial support for the review, and the role of the funders or sponsors in the review. | NA | NA |
| Competing interests | 26 | Declare any competing interests of review authors. | NA | NA |
| Availability of data, code and other materials | 27 | Report which of the following are publicly available and where they can be found: template data collection forms; data extracted from included studies; data used for all analyses; analytic code; any other materials used in the review. | NA | NA |

# PRISMA 2020 for Abstracts checklist

| **Section/topic** | **Item No** | **Checklist item** | **Reported on Page Number/Line Number** | **Reported on Section/Paragraph** |
| --- | --- | --- | --- | --- |
| **TITLE** | | | | |
| Title | 1 | Identify the report as a systematic review. | Page1/line2-3 | Abstract/title |
| **BACKGROUND** | | | | |
| Objectives | 2 | Provide an explicit statement of the main objective(s) or question(s) the review addresses. | Page1/line11-15 | Abstract/Paragraph1 |
| **METHODS** | | | | |
| Eligibility criteria | 3 | Specify the inclusion and exclusion criteria for the review. | Page1/line15-19 | Abstract/Paragraph1 |
| Information sources | 4 | Specify the information sources (e.g. databases, registers) used to identify studies and the date when each was last searched. | Page1/line16 | Abstract/Paragraph1 |
| Risk of bias | 5 | Specify the methods used to assess risk of bias in the included studies. | Page1/line19-20 | Abstract/Paragraph1 |
| Synthesis of results | 6 | Specify the methods used to present and synthesize results. | Page1/line19-21 | Abstract/Paragraph1 |
| **RESULTS** | | | | |
| Included studies | 7 | Give the total number of included studies and participants and summarise relevant characteristics of studies. | Page1/line22 | Abstract/Paragraph1 |
| Synthesis of results | 8 | Present results for main outcomes, preferably indicating the number of included studies and participants for each. If meta-analysis was done, report the summary estimate and confidence/credible interval. If comparing groups, indicate the direction of the effect (i.e. which group is favoured). | Page1/line22-32 | Abstract/Paragraph1 |
| **DISCUSSION** | | | | |
| Limitations of evidence | 9 | Provide a brief summary of the limitations of the evidence included in the review (e.g. study risk of bias, inconsistency and imprecision). | Page2/line32-34 | Abstract/Paragraph1 |
| Interpretation | 10 | Provide a general interpretation of the results and important implications. | Page2/line32-34 | Abstract/Paragraph1 |
| **OTHER** | | | | |
| Funding | 11 | Specify the primary source of funding for the review. | NA | NA |
| Registration | 12 | Provide the register name and registration number. | NA | NA |

# List of literature included in the Meta-analysis

[1] ALBINET C T, ABOU-DEST A, ANDRé N, et al. Executive functions improvement following a 5-month aquaerobics program in older adults: Role of cardiac vagal control in inhibition performance [J]. Biological psychology, 2016, 115: 69-77.

[2] ALBINET C T, BOUCARD G, BOUQUET C A, et al. Increased heart rate variability and executive performance after aerobic training in the elderly [J]. European journal of applied physiology, 2010, 109(4): 617-24.

[3] ANTUNES H K, SANTOS-GALDUROZ R F, DE AQUINO LEMOS V, et al. The influence of physical exercise and leisure activity on neuropsychological functioning in older adults [J]. Age (Dordrecht, Netherlands), 2015, 37(4): 9815.

[4] BLISS E S, WONG R H X, HOWE P R C, et al. The Effects of Aerobic Exercise Training on Cerebrovascular and Cognitive Function in Sedentary, Obese, Older Adults [J]. Front Aging Neurosci, 2022, 14: 892343.

[5] CHAPMAN S B, ASLAN S, SPENCE J S, et al. Shorter term aerobic exercise improves brain, cognition, and cardiovascular fitness in aging [J]. Front Aging Neurosci, 2013, 5: 75.

[6] EDWARDS M K, LOPRINZI P D. Effects of a Sedentary Intervention on Cognitive Function [J]. American journal of health promotion : AJHP, 2018, 32(3): 595-605.

[7] ERICKSON K I, VOSS M W, PRAKASH R S, et al. Exercise training increases size of hippocampus and improves memory [J]. Proc Natl Acad Sci U S A, 2011, 108(7): 3017-22.

[8] GOTHE N P, KESWANI R K, MCAULEY E. Yoga practice improves executive function by attenuating stress levels [J]. Biological psychology, 2016, 121(Pt A): 109-16.

[9] LECKIE R L, OBERLIN L E, VOSS M W, et al. BDNF mediates improvements in executive function following a 1-year exercise intervention [J]. Front Hum Neurosci, 2014, 8: 985.

[10] PREHN K, LESEMANN A, KREY G, et al. Using resting-state fMRI to assess the effect of aerobic exercise on functional connectivity of the DLPFC in older overweight adults [J]. Brain and cognition, 2019, 131: 34-44.

[11] SCHWARTZ B, KAPELLUSCH J M, BACA A, et al. Medium-term effects of a two-desk sit/stand workstation on cognitive performance and workload for healthy people performing sedentary work: a secondary analysis of a randomised controlled trial [J]. Ergonomics, 2019, 62(6): 794-810.

[12] TORBEYNS T, DE GEUS B, BAILEY S, et al. Bike Desks in the Classroom: Energy Expenditure, Physical Health, Cognitive Performance, Brain Functioning, and Academic Performance [J]. Journal of physical activity & health, 2017, 14(6): 429-39.

[13] VOSS M W, HEO S, PRAKASH R S, et al. The influence of aerobic fitness on cerebral white matter integrity and cognitive function in older adults: results of a one-year exercise intervention [J]. Human brain mapping, 2013, 34(11): 2972-85.
